# Effects of Dopamine on Sensitivity to Social Bias in Parkinson's Disease

**DOI:** 10.1371/journal.pone.0032889

**Published:** 2012-03-09

**Authors:** Atbin Djamshidian, Sean S. O'Sullivan, Andrew Lees, Bruno B. Averbeck

**Affiliations:** 1 Sobell Department of Motor Neuroscience and Movement Disorders, Institute of Neurology, University College London (UCL), London, United Kingdom; 2 Department of Molecular Neuroscience and Reta Lila Weston Institute for Neurological Studies, University College London (UCL), London, United Kingdom; 3 Laboratory of Neuropsychology, National Institute of Mental Health, National Institutes of Health, Bethesda, Maryland, United States of America; Centre Hospitalier Universitaire Vaudois (CHUV), Switzerland

## Abstract

Patients with Parkinson's disease (PD) sometimes develop impulsive compulsive behaviours (ICBs) due to their dopaminergic medication. We compared 26 impulsive and 27 non-impulsive patients with PD, both on and off medication, on a task that examined emotion bias in decision making. No group differences were detected, but patients on medication were less biased by emotions than patients off medication and the strongest effects were seen in patients with ICBs. PD patients with ICBs on medication also showed more learning from negative feedback and less from positive feedback, whereas off medication they showed the opposite effect.

## Introduction

The basal ganglia, the prefrontal cortex, the amygdala and the fusiform gyrus are all believed to be involved in face processing [1], [2], [3], [4]. Therefore, pathology in these circuits could lead to deficits in face processing. Deficits in recognizing facial expressions, particularly those expressing anger and fear, have been demonstrated in patients with various kinds of addictions and compulsive behaviours [5], [6]. Results in patients with Parkinson's disease (PD) on the other hand are still inconclusive with some [7], [8], [9], [10], [11] but not all [12] studies showing impairment of facial processing.

In this study we tested PD patients on and off their usual dopaminergic medication to examine the effects of impulsivity and dopamine in decision making in a task relying on emotional cues. Participants were asked to learn by trial and error, which of two faces (a happy and an angry face) was associated with a small monetary reward and then to pick that face as often as possible. In theory the facial expression should not influence decision making, however studies in healthy volunteers showed that subjects would select the happy face more often than the angry one, especially when learning was difficult [13]. This behaviour is called ‘positive emotional bias’. In contrast negative biases in processing emotional information have been found in patients with depression [14]. Both positive and negative emotional biases can lead to irrational decision making which might lead to damaging behaviours, such as overconfidence or depression. From a different perspective, however, the emotion bias may reflect appropriate processing of social communication information, which is important for social function.

A functional imaging study has shown that emotion bias correlates with activation in the anterior cingulate, which is known to have the greatest dopamine innervation in the cortex [15] and the temporo-parietal junction [16]. Thus dopamine and especially unphysiological dopaminergic medication might alter emotion bias. In contrast to the emotion bias, reward feedback information in this task activated the ventral striatum and ventral-medial prefrontal cortex, which was necessary to learn which face was most often being rewarded [16].

Impulsive compulsive behaviours (ICBs) such as pathological gambling and compulsive sexual behaviour are triggered by dopaminergic medication. Although some risk factors for ICBs and behavioural changes on metric tasks have been described [17] it is unclear whether dopamine induced changes in emotional bias are a potential risk factor for impulsivity. ICBs predominate in the “on” state which might suggest that dopaminergic medication changes the value of social feedback, and might reduce emotional bias.

Thus we speculated that PD patients with ICBs (PD+ICB) would be less biased by emotions in their “on” versus “off” state and hypothesised that controls would learn better on this task than PD+ICB patients.

## Methods

### Objectives

To assess the effects of dopaminergic medication on sensitivity to social bias in PD patients with and without ICBs.

### Participants

Twenty five PD patients without ICBs (PD−ICB) and 26 PD+ICB patients were recruited from the National Hospital for Neurology and Neurosurgery, Queen Square, London. All patients fulfilled the Queen Square Brain Bank criteria for the diagnosis of PD [18] and were taking L-dopa. PD+ICB patients were diagnosed using proposed criteria [19], [20], [21], [22]. Many PD+ICB patients had more than one ICB (see Table 1). L-dopa equivalent units (LEU) of patients' regular daily dopamine replacement therapies were calculated as described elsewhere [22]. All patients showed a significant improvement (>30% improvement) after dopamine replacement therapy intake on the UPDRS (part 3) motor score. Sixteen healthy elderly volunteers matched for age, gender and education were recruited, usually from amongst the patient's spouses or partners. Patients who scored under 26/30 points on the Mini Mental State Examination (MMSE) [23] were excluded from this study.

**Table 1:** Demographic characteristics.

	Controls (1)	PD+ICB (2)	PD−ICB (3)	t value, χ^2^ and F-value	p-value; columns
Participants(no.)	16	26	27		
Age (yrs)	59.1±11.8	58.0±9.3	65.3±5.3	F = 5.2	0.008*;(1,2,3)
Gender (male)	14	22	24	κ^2^ = 5.9	0.3; (1,2,3)
At PD onset (yrs)		47.7±9.5	55.3±7.4	t = 3.28	0.002*;(2,3)
PD Disease duration (yrs)		11.0±4.2	10.0±6.6	t = 0.52	0.48;(2,3)
Education (yrs)	13.1±3.2	13.1±2.8	14.7±2.5	F = 2.4	>0.09; (1,2,3)
LEU dose(mg/day)		934.2±407	740.1±369	t = 1.8	0.072;(2,3)
PD patients currently using DA		13/26	21/27	κ^2^ = 5.1	0.024*;(2,3)
UPDRS on		16.2±10.6	21.1±9.0	t = 1.7	0.09;(2,3)
UPDRS off		31.0±11.3	32.1±10.6	t = 0.5	0.6;(2,3)
Improvement in UPRDS (%)		47.7	34.2		
Hypersexuality		12			
PG		13			
Punding		7			
Shopping		5			

UPDRS = Unified Parkinson's Disease Rating Scale; LEU = L-dopa equivalent units; DA = dopamine agonists. All values are mean ± SD. Significant differences are labelled with “*”. P-values refer to columns indicated in brackets. Controls (column 1), PD+ICB (column 2), PD−ICB (column 3).

### Description of Procedures

PD patients were tested before and after their usual anti-Parkinson medication in a counterbalanced sequence to account for order effects. All patients who were tested in their “off medication state” did not take their usual anti-Parkinson medication, including both L-dopa and any dopamine agonists, for at least 12 hours and performed the task between 8.00am and 9.00am. They were then retested in their “on medication” state the following day, usually mid mornings. Those patients who were tested “on medication” first performed this task usually in mid-morning when their motor symptoms were well controlled. They were re-visited on the following day prior to their medication for the second test. Results were compared with 16 controls who were matched for age to the PD+ICB group and who were tested over two days but did not take any anti-Parkinson medication. At the end of the study all participants received a modest honorarium depending on their final score (usually £5–£10).

### Face decision task

**Figure 1:**
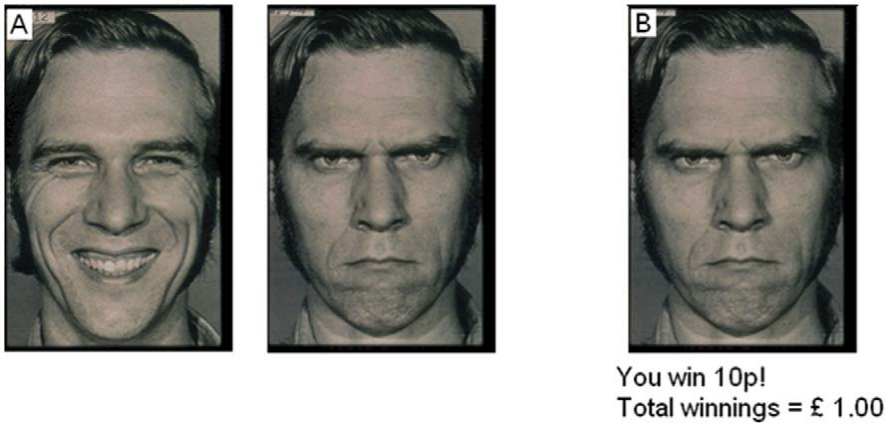
Face task. A: One happy and one angry face were presented side by side on a black screen. B: After a choice (in this case angry face) visual and acoustic feedback was given immediately.

We used a probabilistic face decision task, which has been described previously [13]. Participants performed the task on a laptop computer either at home or in a quiet room to minimize distractions. They were told to choose between 2 stimuli (a happy and an angry face) which were presented side by side on a black screen and had different probabilities of being rewarded (Figure 1). Feedback was given immediately and correct choices were rewarded with 10 pence whereas incorrect choices resulted in no reward. In total participants performed 4 blocks consisting of 26 trials per block and were told to maximize their winnings by picking the rewarded picture as many times as possible. The reward probabilities across the 4 blocks were kept constant (60∶40) and were mapped to each emotion in a balanced manner, such that the happy face was the high probability stimulus in 2 blocks and the angry face in 2 blocks. Two identities were also used in interleaved blocks. Feedback was given stochastically, i.e. after each selection, rewards were delivered pseudo-randomly with a fixed probability that depended on the face and the block [13]. Emotion bias was determined by comparing how often, when participants do not select the most often rewarded face, they select the happy face as opposed to how often they select the angry face. Heuristically, one can also ask, given equivalent reward feedback for both faces, how often are participants selecting the happy face? If it's well over 50% of the time, then they are biased towards the happy face.

### Ethics

All participants provided written informed consent according to the declaration of Helsinki and the study was approved by the UCLH Trust ethics committee.

### Statistical methods

All data analysis was carried out in Matlab. Details about the statistical analysis have been reported elsewhere [13], [24]. Briefly, we fit a statistical model known as an ideal observer, which tracked positive and negative feedback given for each face, and made an optimal decision about which face should be chosen for each trial. The model made its decision based on only the reward feedback, so it was not biased by the emotional content of the face. The choices of the participants could then be compared to the optimal choices of the model, which were based only on reward feedback.

Analysis of variance was carried out on emotion bias, overall learning, and the effects of positive vs. negative feedback. In all cases, these parameters were computed for each subject, and then an ANOVA was run with the corresponding behavioural measure as a dependent variable. Emotion bias and overall learning were characterized by compiling a 2×2 contingency table for each subject. Each cell in the contingency table contained counts of choice patterns of the model and the observer. Based on the counts in the table, emotion bias was computed as:
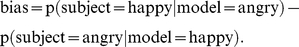
(1)Reward learning was correspondingly given by:
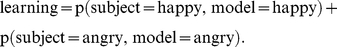
(2)


## Results

### Demographic and clinical features

Across groups there was a significant effect of age (F_(2,66)_ = 5.3, p = 0.008). Post hoc analysis showed that the PD−ICB group was older than the PD+ICB group (p = 0.009). There was no difference between controls and PD or PD+ICB patients (p>0.08). PD+ICB patients had a significantly younger disease onset relative to PD−ICB patients (t_52_ = 3.28, p = 0.002). There was no difference in LEU dose, disease duration or years of education between both patient groups. Further there was no group difference in UPDRS part 3 motor scores prior to (‘off-state’) and similarly no group difference after medication (‘on-state’). Significantly more PD−ICB than PD+ICB patients were treated with a dopamine agonist, which is in line with accepted clinical guidelines of managing an ICB in PD (see table 1).

### Face decision task

**Figure 2:**
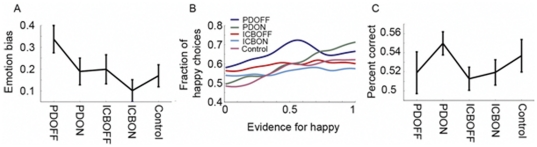
Behavioral results. All error bars are 1 s.e.m. A. Emotion bias for each group. B. Evidence vs. choice curve showing increased selection of happy faces when evidence supports angry face. C. Percent correct for each group.

We first examined the PD−ICB and PD+ICB groups on and off medication, excluding controls, with respect to emotion bias (Fig. 2A,B) and overall reward learning (Fig. 2B,C). Emotion bias is defined as choosing the happy face when they should choose the angry face vs. choosing the angry face when they should choose the happy face. Complementing this, reward learning is defined as the proportion of times that participants chose the angry face when they should have chosen the angry face (where “should” is estimated using the ideal observer model) and the number of times participants chose the happy face when they should have chosen the happy face (see methods). Thus, these two measures assess complementary aspects of the decision process.

We first examined just the PD groups off and on medication. We found that dopamine medication decreased the emotion bias across groups (F_1, 48_ = 6.29, p = 0.016), thus all patients chose the angry picture more often in their ‘on’ compared to their ‘off’ state. There was no difference between groups in emotion bias (F_1, 48_ = 2.57, p = 0.115) and no interaction between medication and group (F_1, 48_ = 0.24, p = 0.627). Further, there was no effect of fraction correct performance (reward learning), when included as a covariate, on emotion bias (F_1, 48_ = 0.16, p = 0.689). Thus, participants who had a larger emotion bias did not necessarily learn less. When the PD groups were analysed individually to examine medication effects, there was a significant decrease in emotion bias in the PD group on vs. off medication (F_1, 24_ = 4.56, p = 0.043) but not in the ICB group (F_1, 25_ = 2.48, p = 0.128). In contrast to the emotion bias, there were no effects of medication (F_1, 49_ = 1.65, p = 0.205) or group (F_1, 49_ = 1.31, p = 0.257) on overall learning (Fig. 2C).

We next compared the PD−ICB and PD+ICB groups, off and on medication, to controls, pair-wise. However, there were no significant group differences in emotion bias (p>0.071). Following this, we carried out planned comparisons on emotion bias separately in each group, to see if each group was significantly affected by the facial expression (Fig. 2B). A significant positive emotion bias indicates that a group is significantly biased by the emotion expression, choosing happy more than angry, given equivalent reward feedback. There were significant effects of emotion bias in the PD−ICB group on medication (t_24_ = 3.09, p = 0.005), off medication (t_24_ = 5.37, p<0.001) and ICB group off medication (t_25_ = 2.98, p = 0.006) and controls (t_15_ = 3.36, p = 0.005). The PD+ICB on medication group just missed significance (t_25_ = 2.05, p = 0.051). For overall learning performance, the PD−ICB on (t_24_ = 4.11, p<0.001) and control (t_15_ = 2.17, p = 0.048) groups were above chance, but all other groups were at chance (p>0.156).Thus, all patients, except the PD+ICB on group, chose the happy face significantly more often than the angry face, given equivalent reward feedback.

**Figure 3:**
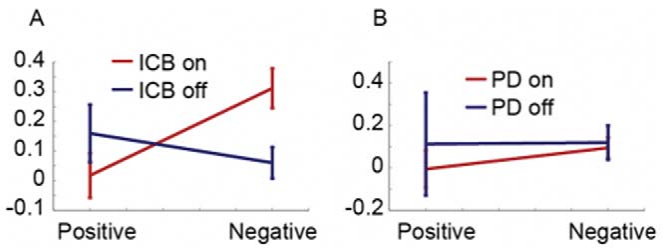
Learning from positive vs. negative feedback. Error bars show 1 s.e.m. A. Parameter values from the Bayesian model in PD+ICBs. B. Parameter values in PDs.

In the final analysis we split overall learning into parameters which separately measured learning from positive and negative feedback in the PD groups off and on medication (Fig. 3). Results from this analysis have to be interpreted with caution, as the groups generally did not show robust learning, measured as choices which were consistent with the ideal observer, except the PD−ICB patients on medication. It is still possible, however, that the participants were responding to positive and negative feedback, without integrating it effectively over trials. We found that in the PD group there were no effects of medication on learning from positive vs. negative feedback (F_1, 24_ = 0.23, p = 0.629) or medication by feedback type interactions (F_1, 24_ = 0.1, p = 0.753). However, in the PD+ICB group we found that there was a significant medication by feedback type interaction (F_1, 25_ = 7.35, p = 0.008) but no main effect of medication (F_1, 25_ = 0.57, p = 0.452). Thus, consistent with our previous study [25], increased dopamine levels in the PD+ICB group increased sensitivity to negative feedback and decreased sensitivity to positive feedback.

## Discussion

All patients chose the happy face more than the angry face, when expressions were matched for reward history, in their ‘off’ versus ‘on’ state. Furthermore, when examined pair-wise there were no differences in emotion bias or learning between the patient groups and the control group. This might be because participants generally were not able to learn and integrate the reward information effectively, since their behaviour did not correlate well with the ideal observer, which is a purely statistical model of how one should learn. When each group was analysed individually, we found that all groups, other than the PD+ICB group ‘on medication’, had a significant emotion bias and preferred the happy to the angry face. Our finding is consistent with previous studies using the same task [16], [24], [26]. Thus, dopaminergic medication in PD appears to decrease sensitivity to social bias in decision making. This reduction of emotional bias ‘on medication’ is particularly interesting since it has been reported that the processing of negative emotions is impaired in PD [27] and improves after dopaminergic medication [9], [28]. In our study however PD patients in general and particularly PD+ICB patients picked the angry picture more often in their ‘on’, rather than ‘off’ state. The lack of emotional bias in PD+ICB patients suggests that these patients might be prepared to ignore negative feedback in the form of an angry face more easily when associated with a potential short term reward. It is also possible that this shift away from emotional biases could mean that PD+ICB patients care less about other's emotions, reflecting reduced empathy in the patients. Several of the PD+ICB patients spontaneously reported during testing that they *‘anticipated’* the correct choice. This anticipation may be mediated via the ventral striatum and anticipation of a conflict is processed via the anterior cingulate [29], [30]. This type of response has also been found in another task in which participants could control the amount of evidence they gathered before making a decision [31].

Thus, PD+ICB patients may learn poorly in some conditions because they might use irrational estimates of correct responses, rather than trying to learn from previous trials.

We found that both patient groups selected angry faces less than controls, although none of the groups differed significantly from controls. Patients with schizophrenia also select angry faces less often than controls, given equivalent reward evidence [26]. In our study, increasing dopamine levels decreased this emotional bias in both PD groups. This would be consistent with the hypothesis that effects seen in the study of patients with schizophrenia were driven more by their medication status than by a possible increase in striatal dopamine levels [32], [33].

The nonapeptide oxytocin can decrease emotion bias in this task, specifically by increasing choices of angry faces when the evidence supports angry faces [24]. Interestingly, the results in the present study tended to be consistent across levels of evidence for each face. One could have hypothesized that dopamine modulation may have affected learning from reward feedback which has been found in some [34], [35] but not all studies in PD [36], without affecting social processing [24]. We found effects of dopamine on learning in this study, consistent with our previous results [25]. Work in rodents has shown consistently that dopamine and oxytocin can interact [37] and in humans oxytocin can increase generosity, possibly by inducing striatal dopamine release [38] suggesting that dopamine and oxytocin may interact to determine the effects of social stimuli on behaviour. Of note dopamine increases altruistic punishment only in PD+ICB patients but not in non-impulsive PD patients [39]. Similarly oxytocin can increase punishment behaviour towards competing groups [40].

In an fMRI study of this task, larger modulation of blood-oxygen-level dependent (BOLD) responses by reward prediction error in the anterior cingulate and temporal parietal junction, were correlated with smaller emotion bias effects. Further those subjects that learned best in this task had the smallest modulation of BOLD responses by reward prediction error [16]. Thus, learning and increased BOLD modulation by reward prediction error appear to be inversely correlated. Therefore the decreased social bias seen in this study may be driven by increased reward prediction error processing in the anterior cingulate and/or the temporo-parietal junction. In previous work on the Stroop task, which is likely mediated by the anterior cingulate as well [41], we have found that PD−ICB and PD+ICB patients performed similarly at a group level, but medication improved performance in both groups, as well as improving cognitive flexibility [42]. This is consistent with the hypothesis that dopamine in the anterior cingulate is mediating the effects we have found in this study.

In summary, dopaminergic medication decreased sensitivity to social bias in both groups of PD patients, in the absence of group effects and none of the groups differed from matched controls. Our results might imply that PD+ICB patients ‘on medication’ are less affected by emotional feedback, which could be partly responsible for their difficulties integrating into society and following social norms. This is of interest in relation to the finding of increased schizotypy in PD with ICBs [43]. We also found effects of dopamine on the balance between learning from positive vs. negative feedback in the PD+ICB group, but not in the PD−ICB group.
